# The Early Endocytosis Gene *PAL1* Contributes to Stress Tolerance and Hyphal Formation in *Candida albicans*

**DOI:** 10.3390/jof9111097

**Published:** 2023-11-10

**Authors:** Miranda Yu, Dakota Ma, Susan Eszterhas, Christiane Rollenhagen, Samuel A. Lee

**Affiliations:** 1 Thayer School of Engineering at Dartmouth, Dartmouth College, Hanover, NH 03755, USA; miranda.j.yu.24@dartmouth.edu; 2 Medicine Service, White River Junction VA Medical Center, Hartford, VT 05009, USA; dakota.c.ma.22@gmail.com (D.M.); susan.k.eszterhas@dartmouth.edu (S.E.); 3 Department of Biological Sciences, Dartmouth College, Hanover, NH 03755, USA; 4 Department of Medicine, Geisel School of Medicine, Dartmouth College, Hanover, NH 03755, USA; rollen72@stanford.edu

**Keywords:** *Candida albicans*, clathrin-mediated endocytosis, stress tolerance, hyphal formation

## Abstract

The endocytic and secretory pathways of the fungal pathogen *Candida albicans* are fundamental to various key cellular processes such as cell growth, cell wall integrity, protein secretion, hyphal formation, and pathogenesis. Our previous studies focused on several candidate genes involved in early endocytosis, including *ENT2* and *END3*, that play crucial roles in such processes. However, much remains to be discovered about other endocytosis-related genes and their contributions toward *Candida albicans* secretion and virulence. In this study, we examined the functions of the early endocytosis gene *PAL1* using a reverse genetics approach based on CRISPR-Cas9-mediated gene deletion. *Saccharomyces cerevisiae* Pal1 is a protein in the early coat complex involved in clathrin-mediated endocytosis that is later internalized with the coat. The *C. albicans pal1*Δ/Δ null mutant demonstrated increased resistance to the antifungal agent caspofungin and the cell wall stressor Congo Red. In contrast, the null mutant was more sensitive to the antifungal drug fluconazole and low concentrations of SDS than the wild type (WT) and the re-integrant (KI). While *pal1*Δ/Δ can form hyphae and a biofilm, under some hyphal-inducing conditions, it was less able to demonstrate filamentous growth when compared to the WT and KI. The *pal1*Δ/Δ null mutant had no defect in clathrin-mediated endocytosis, and there were no changes in virulence-related processes compared to controls. Our results suggest that *PAL1* has a role in susceptibility to antifungal agents, cell wall integrity, and membrane stability related to early endocytosis.

## 1. Introduction

The opportunistic fungus *Candida albicans* is a major cause of severe invasive infections in hospitalized patients that may lead to substantial morbidity and mortality. A more complete understanding of the mechanisms of invasive candidiasis is needed for the development of more effective diagnostic and therapeutic strategies. One major contributor to *Candida* pathogenesis is secretion. This process is needed for fundamental biological and virulence-related processes, such as filamentation and biofilm formation [1,2]. It has been suggested that endocytosis is another cellular process that contributes to virulence by allowing cells to uptake not only nutrients but also signaling molecules, regulate plasma membrane structure, and maintain cell wall composition and integrity [3]. These functions are crucial to virulence-related processes that include filamentation, biofilm formation, and secretion of virulence-associated proteins such as aspartyl proteases [4,5]. Several studies of endocytic and secretory mutants support the connection between yeast intracellular transport pathways and pathogenesis. The secretory mutants *vps1*, *vps4*, and *pep12* in *C. albicans* have marked abnormalities in endocytic and secretory functions, as well as in filamentation and pathogenesis [6,7,8]. Furthermore, our previous work with the *C. albicans* early endocytosis genes *ENT2* and *END3* also demonstrated endocytic defects in null mutants coinciding with reduced protease secretion, impaired filamentation, biofilm formation, and decreased virulence traits [9,10].

Among other endocytic processes, clathrin-based endocytosis (CME) is well characterized in yeast and is a significant type of endocytosis in both *C. albicans* and the model yeast *Saccharomyces cerevisiae* [11]. More than 50 proteins enable cargo to be transported intracellularly in a highly organized and sequential process [3]. Endocytosis starts at the formation of the initial endocytic site, which is defined by cortical actin patches that are subsequently accompanied by assembly processes for vesicle formation [2]. Several coat proteins facilitate the formation of a clathrin-coated transport vesicle that moves from the donor to target membranes in the delivery process. The early-coat proteins determine the locations of endocytic sites and recruit cargo. They consist of clathrin, the AP-2 adaptor protein, Ede1p, Syp1p, and Pal1p, which are recruited in a well-orchestrated manner [12]. Clathrin, AP-2, and Pal1p are internalized with the clathrin coat, while Ede1p and Syp1p remain associated with the donor plasma membrane [13]. Subsequent middle- and late-coat proteins drive actin assembly and membrane invagination, resulting in mature endocytic vesicle formation and scission [2,14]. Sla2p is important for the transition to middle-coat endocytosis, and is joined by the epsins Ent1p and Ent2p, as well as the clathrin-binding proteins Yap1801p and Yap1802p, which are involved in clathrin cage assembly [15,16]. Finally, late-coat proteins such as Pan1p, Sla1p, Prk1p, End3p, and Las17p form a stable transport complex before mobile endocytosis begins with the WASP/Myo, amphiphysin, and actin modules [17,18,19,20,21].

Several genes encoding key coat proteins, including *EDE1*, *SLA2*, *PAN1*, *SLA1*, *END3*, and *LAS17*, have been studied in detail [2]. While *EDE1* was found to be non-essential for *C. albicans* growth and filamentation, the other endocytic genes appear to be essential for filamentation and hyphal growth, especially *Ca WAL1*, which is the *C. albicans* homolog of the late-coat protein-encoding gene *LAS17* [22,23]. The early-coat protein Pal1p has yet to be studied in *C. albicans* but is orthologous to Pal1p in *S. cerevisiae*. Though its molecular function has not yet been fully clarified, Pa1lp localizes to the cell periphery and the endocytic sites of the bud neck during early endocytosis in *S. cerevisiae*. Pal1p interacts with the early-coat protein Ede1p and the middle-coat protein Slap2 [12,24].

Due to its involvement in the early stage of the clathrin-mediated endocytosis pathway, we examined *C. albicans PAL1* to determine its roles in endocytosis and pathogenesis; specifically, protease secretion, filamentation, and biofilm formation. We also investigated the role of *PAL1* in antifungal sensitivity to shed light on any clinical implications. Our studies examine how the early-coat protein Pal1p contributes to establishing endocytic pathways fundamental to *Candida albicans* invasiveness and infection.

## 2. Materials and Methods

### 2.1. Identification of the C. albicans Ortholog of PAL1

The DNA and protein sequences of *S. cerevisiae PAL1* (YDR348C) were retrieved using the Saccharomyces Genome Database (http://www.yeastgenome.org (accessed on 1 June 2020)). The predicted DNA and protein sequences for the *S. cerevisiae PAL1* ortholog were identified as the uncharacterized ORF *C3_01890C* in *C. albicans* in the Candida Genome Database (http://www.candidagenome.org (accessed on 1 June 2020)). SnapGene Version 7.0.2 (http://www.snapgene.com (accessed on 2 June 2020)) was used to align the two protein sequences by the Smith–Waterman method [25]. Protein domain identification and annotation was conducted through the Simple Modular Architecture Research Tool (SMART), a web resource updated in 2020 that is available at https://smart.embl.de (accessed on 3 April 2023) [26].

### 2.2. Deletion of C. albicans Pal1

The *C. albicans pal1*Δ/Δ null mutant (KO) and re-integrant (KI) were generated from the AHY940 parental strain following the CRISPR-Cas9 protocol established by the Hernday laboratory [27]. The primers used for these gene mutations are indicated in Table 1.

### 2.3. Preparation of Genomic DNA and Plasmid Isolation

Table 2 indicates the plasmids generated and utilized in this study. Genomic DNA extraction from yeast cells was conducted according to the manufacturer’s instructions using the MasterPure Yeast DNA purification kit (Epicentre Biotechnologies, Madison, WI, USA). Plasmids were transformed and maintained in competent *Escherichia coli* DH5α cells (Invitrogen, Waltham, MA, USA), then extracted with the Qiagen Plasmid Miniprep system protocol (Qiagen, Germantown, MD, USA) using overnight cultures of transformed *E. coli* cells grown at 37 °C in LB medium (1% tryptone, 0.5% glucose, and 1% NaCl) with 100 μg/mL ampicillin.

### 2.4. Strains, Media, and Cell Culture

Table 3 lists the *C. albicans* strains utilized in this study. Unless otherwise stated, strains were cultured in YPD (1% yeast extract, 2% peptone, 2% glucose) or in YNB minimal media (0.67% yeast nitrogen base, amino acids, 2% glucose) and incubated at 30 °C with shaking at 250 rpm. Solid media with 2% agar were prepared for agar plate assays. Inoculated *C. albicans* plates were incubated at either 30 °C or 37 °C.

The human vaginal keratinocyte cell line VK-2/E6E7 (ATCC CRL-2616, Manassas, VA) was grown in Keratinocyte-SFM media (Invitrogen, Waltham, MA, USA) in a cell culture incubator at 37 °C with 5% CO_2_ with cells fed every two days and split once a week according to the manufacturer’s instructions. 

### 2.5. Cell Growth Assay and Assays for Response to Environmental Stress and Filamentation

To determine the effect of *PAL1* on cell growth, *C. albicans pal1*Δ/Δ or control (wild-type and re-integrant) strains were first grown in YPD overnight at 30 °C and counted. After the strains were diluted in YNB to 1 × 10^6^ cells/mL, 100 µL of cell dilution was loaded in 96-well plates in triplicate, with three replicates per strain. A BioTek Synergy H1 microplate reader (Agilent Technologies, Santa Clara, CA, USA) provided the 30 °C growth rates by recording OD_600 nm_ extinction at 30 min intervals over 16 h. The average OD_600_ values over time were graphed using Excel Version 16.66.1 (Microsoft Corporation, Redmond, WA, USA) to display the growth curve, and the data were analyzed.

As previously described [6,8], agar plate assays also qualitatively described growth rates. After growing cell cultures overnight in YPD at 30 °C, the cells were counted and diluted to 1 × 10^8^ cells/mL concentrations in YPD for this and other agar plate assays. Five 5-fold serial dilutions produced five concentrations where 5 μL of each suspension was spotted onto agar plates. Growth on YPD plates was assessed after incubating the plates at 30 °C, 37 °C, and 42 °C for 48 h. Response to cell wall stressors was assessed in triplicate for each strain after 48 h at 30 °C on YNB plates containing 100 µg/mL Calcofluor White or 140 µg/mL Congo Red, and on YPD plates containing 0.02% SDS (all Sigma-Aldrich, St. Louis, MO, USA). To assay for antifungal drug sensitivity, YPD agar plates containing fluconazole (1, 2, and 4 µg/mL), caspofungin (0.025, 0.05, and 0.1 µg/mL), and amphotericin B (0.11, 0.33, and 1 µg/mL) were prepared, and growth on each type of media at three concentrations at 30 °C was assessed after 24 h in triplicate for each strain (all Sigma-Aldrich, St. Louis, MO, USA).

In addition, growth was observed over 24 h in a liquid YPD medium assay with each of the three antifungal drugs: fluconazole, caspofungin, and amphotericin B. Cell cultures were grown overnight in YPD at 30 °C, then counted and diluted to final concentrations of 1 × 10^6^ cells/mL in liquid YPD media containing fluconazole, caspofungin, and amphotericin B for final concentrations of 2 µg/mL, 0.05 µg/mL, and 0.33 µg/mL, respectively. A BioTek Synergy H1 microplate reader recorded OD_600 nm_ extinction at 30 min intervals over 24 h in 96-well plates where 100 µL of cell dilution was loaded in triplicate, with three replicates for each strain. The average OD_600_ values over time were graphed using Excel (Microsoft Corporation, Redmond, WA, USA) to display the growth curve.

Filamentation was visualized after incubating YPD plates with 10% (vol/vol) fetal calf serum (FCS, Fisher Scientific, Waltham, MA, USA), Medium 199 supplemented with L-glutamine (M199, Sigma-Aldrich, St. Louis, MO, USA), RPMI-1640 at pH 7.0 (Fisher Scientific, Waltham, MA, USA), and Spider medium as previously described [28] at 37 °C for 72 h in triplicate for each strain.

### 2.6. Fluorescence Microscopy of C. albicans Strains

We visualized cell morphology of the yeast and hyphal forms with slight modifications to the methods described previously [10,29]. Yeast growth was conducted with standard overnight cell cultures in liquid YPD at 30 °C. Filamentous growth was induced by overnight incubation in liquid RPMI-1640 media at 37 °C followed by shaking the next day. At 2 and 6 h, cells from the RPMI culture were washed with PBS and mixed in a 2:1 ratio with Calcofluor White (Sigma-Aldrich, St. Louis, MO, USA). Glass slides with the Calcofluor White cell dilution were viewed on a Zeiss Axio Imager M1 fluorescent microscope (Carl-Zeiss AG, Oberkochen, Germany) with a 63× objective lens through both the differential interference contrast (DIC) channel and the 4′,6-diamidino-2-phenylindole (DAPI) filter.

For the cultures incubated for 6 h in RPMI-1640, 100 cells of each strain were counted by morphotype (yeast, hyphae, pseudohyphae) to qualitatively assess for statistical significance in any differences in morphology between the WT or KI and the KO. Contingency table analysis was performed on percentage frequency within strains using a generalized linear model (GLM). Mean differences between cells were obtained by the least square method.

### 2.7. Analyses of Endocytosis with FM4-64

The actively endocytosed lipophilic membrane dye N-(3-triethylammoniumpropyl)-4-(6-(4-(diethylamino)phenylhexatrienyl) pyridiniumdibromide (FM4-64, EMD Millipore, Temecula, CA, USA) was used to assay membrane-related endocytosis and vacuole morphology as described previously [10]. Ice-cold cell cultures grown to the exponential phase were incubated with FM4-64 at a 2 mM concentration for 20 min on ice for dye uptake. The cells were then washed in ice-cold YPD, and subsequently incubated for 5, 15, and 30 min at room temperature. At each time point, a cell aliquot was maintained in ice cold 12 mM sodium azide (Sigma-Aldrich, St. Louis, MO, USA) to halt membrane transport so the uptake process could be observed over time. Vacuolar staining was observed using a Zeiss Axio Imager M1 fluorescent microscope using standard Texas Red filters to visualize FM4-64 dye. The presence and rate of vacuolar membrane staining was used as a representation for endocytosis in image analysis. 

### 2.8. Analysis of Biofilm Formation in C. albicans Endocytosis Mutants

The XTT reduction assay was used to assess biofilm metabolic activity according to previous methods [30]. To induce biofilm growth, overnight cell cultures of *C. albicans pal1*Δ/Δ or control (wild-type and re-integrant) strains in YPD were diluted to a concentration of 1 × 10^6^ cells/mL in liquid RPMI-1640. In total, 100 µL of cell dilution was loaded into a CellBIND 96-well microplate (Corning Inc., Corning, NY, USA) in triplicates for each strain, then incubated at 37 °C for 48 h. Biofilms were washed with PBS then incubated with the XTT-menadione substrate at 37 °C for 2 h before the supernatant was transferred to a new CellBIND 96-well plate. Absorbance at a wavelength of 490 nm was read using the BioTek Synergy H1 microplate reader (BioTek, Winooski, VT, USA) to represent biofilm metabolic activity. Student’s *t*-test in Excel (Microsoft) was used to analyze statistical significance of any differences in absorbance. Biofilms were also visualized using light microscopy on a Nikon Eclipse Ti inverted microscope (Nikon Instruments, Melville, NY, USA and Tokyo, Japan). 

### 2.9. Immunofluorescence Microscopy of Co-Cultured VK-2 Cells with C. albicans Strains

VK-2 cells in keratinocyte serum-free media (SFM) were grown on glass coverslips at 37 °C and 5% CO_2_. They were infected with *C. albicans pal1*Δ/Δ or control strains at an MOI of 0.01 for 6 and 24 h as described [10,31]. VK-2 cells were stained with a 1:100 dilution of a E-cadherin primary antibody (R&D, Minneapolis, MN, USA) in 0.2% gelatin PBS for 1 h, then washed and incubated for 1 h with an anti-rat Alexa Fluor^TM^ 488 secondary antibody (Invitrogen, Waltham, MA, USA). The coverslips were mounted on slides with an antifade mounting solution containing DAPI for nuclear visualization. A Zeiss Axio Imager M1 microscope using standard EGFR and DAPI filters was used to take images and assess for the presence of E-cadherin. 

### 2.10. Western Blotting and Detection of E-Cadherin

Western blotting was performed with a standard protocol as previously described [32] using protein lysate from VK-2 cells infected with *C. albicans pal1*Δ/Δ mutant or control strains in keratinocyte SFM media for 6 h and 24 h at 37 °C and 5% CO_2_. The blot was hybridized overnight in 1x Tris Buffered Saline Casein Blocking Buffer (Bio-Rad, Hercules, CA, USA) containing a 1:500 dilution of a E-cadherin primary antibody (R&D, Minneapolis, MN, USA). The loading control was a 1:1000 dilution of a tubulin antibody (Invitrogen, Waltham, MA, USA). The blot was incubated after washing with an anti-mouse-HRP secondary antibody (Invitrogen, Waltham, MA, USA) for 1 h. The Clarity Max Western ECL substrates (Bio-Rad, Hercules, CA, USA) and the ChemiDoc Imaging System (Bio-Rad, Hercules, CA, USA) were used to perform protein detection and imaging. 

### 2.11. Live/Dead Viability Assay

VK-2 cells were grown in keratinocyte serum-free medium (SFM) in 96-well plates to a 30% confluence and were co-cultured for 4 and 24 h with overnight cultures of *C. albicans* strains grown in YPD at a concentration of 5 × 10^5^ cells/mL. The Invitrogen Live/Dead viability assay (Waltham, MA, USA) was conducted following the manufacturer’s protocol. The Live/Dead data were analyzed using Excel (Microsoft).

## 3. Results

### 3.1. Identification and Comparison of S. cerevisiae PAL1 and C. albicans C3_01890C

The Candida Genome Database has annotated the *C. albicans* gene *C3_01890C* as an ortholog to *S. cerevisiae PAL1* (*YDR348C*) [33]. *C. albicans C3_01890C* is an uncharacterized gene located on chromosome 3 that spans 1888 bp. The transcription unit is split into two exons together encoding a 443 amino acid product. Protein sequence comparisons between *S. cerevisiae PAL1* and *C. albicans C3_01890C* via SnapGene showed modest alignment, with 33.08% identity and 43.23% similarity. Given its moderate degree of homology to *S. cerevisiae* *PAL1*, *C. albicans C3_01890C* was referred to as *PAL1*.

The two alleles of *PAL1* are highly homologous. The nucleotide sequence of the coding exons of the alleles are conserved to the 99.77% level. The three alterations (of the 1332 nucleotides) encode one silent and two similar amino acid substitutions. The introns (529 nucleotides) differ by only 7 nucleotides, including a gap of 3 nucleotides, and the promoter proximal 2 kb has 99.10% sequence identity.

**Figure 1:**
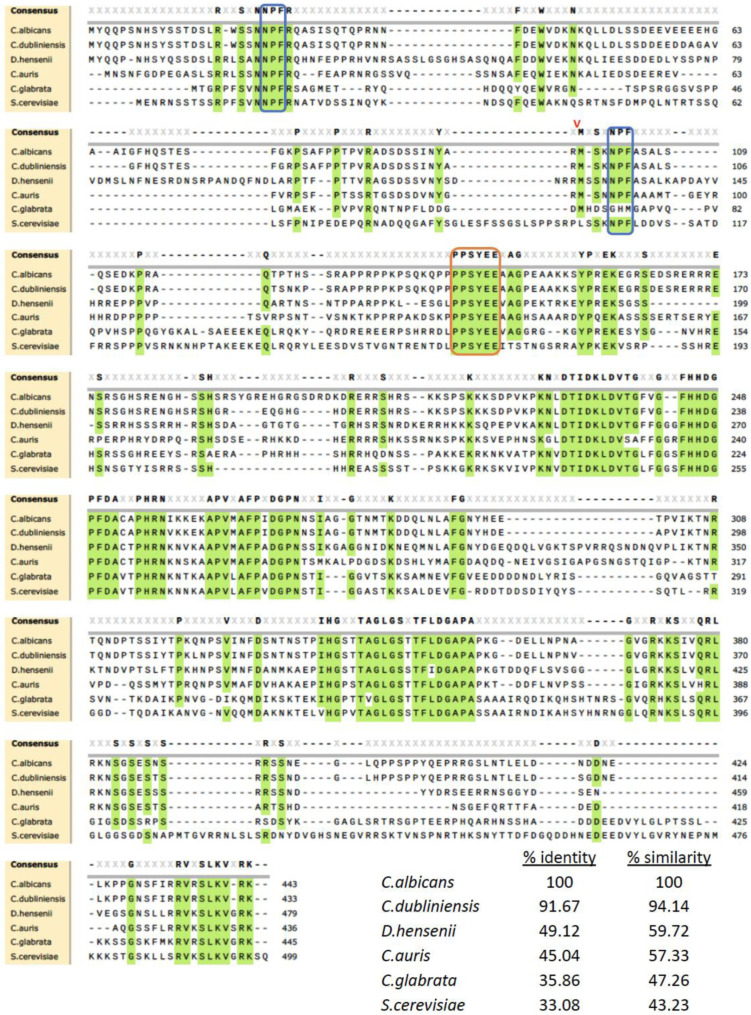
Protein sequence alignments between *C. albicans C3_01890C*, *S. cerevisiae YDR348C* (*PAL1*), and orthologs in three other species of *Candida* and one in the *Debaryomyces* species. The alignment was produced using data from the Candida Genome Database. Regions of amino acid conservation at >80% are highlighted in green. Among the highly conserved sequences are NPF motifs that bind EH domains (boxed in blue), and many proline residues. The sequences in the orange box are a conserved region that are weakly alpha-helical. The red V marks the splice junction in *C. albicans* that is conserved in many but not all the orthologs examined.

Protein sequences of five other fungi species, including three other Candida species, were aligned with *C. albicans C3_01890C* and *S. cerevisiae PAL1* using Muscle 5 on Snapgene to demonstrate other evolutionary relationships (Figure 1). The intron–exon architecture is maintained in most but not all of the *Candida PAL1* orthologs examined. Exceptions include *C. glabrata* as well as *D. hansenii* and *S. cerevisiae*. Relatively low amino acid conservation across species was detected in the first exon, with the notable exception of the conserved NPF motif (boxed in blue) near the N-terminal of the proteins. A second NPF motif was found at the beginning of the second exon. These motifs are bound by the EH (Ets Homology) domains of companion proteins to mediate critical events in endocytosis [34]. Regions of higher conservation are clustered in exon two (starting with methionine 99 in *C. albicans*; marked with a red V in Figure 1). Amino acids 140–145 (PPSYEE; orange box in Figure 1) are well conserved; this region is predicted to have a modest tendency to form an alpha-helical arrangement. Other conserved regions are not predicted to have a specific secondary structure. Also notable are the many well-conserved prolines which may function to delimit domains.

In addition, further comparisons with ten other fungal species, including eight other *Candida* species (three of which are shown in Figure 1), also found notable regions of cross-species conservation. The additional species included *C. tropicalis*, *C. parapsilosis*, *C. orthopsilosis*, *C. guilliermondii*, and *L. elongisporus*. Several areas, including residues 231 to 238, residues 245 to 252, and residues 354 to 357, were absolutely conserved, suggesting functional importance. Interestingly, prolines (P) 140, 141, 249, 265, 270, 274, and 356 were also absolutely conserved across all species. The *PAL1* homolog is also found broadly in other species, such as in *Schizosaccharomyces pombe* and *Aspergillus nidulans*, among others [33].

However, UniProt and SMART annotation did not indicate any documented protein domains in either *S. cerevisiae PAL1* or the protein product of *C. albicans C3_01890C*. Therefore, structural analysis of *C. albicans C3_01890C* provided little additional insight into its molecular function.

### 3.2. Construction of C. albicans pal1Δ/Δ Mutant and Re-Integrant Strains

We next studied the function of *C. albicans PAL1* using a reverse genetics approach. We generated the *C. albicans pal1*Δ/Δ null mutant (KO) and the corresponding re-integrant (KI) strain using a *C. albicans*-adapted CRISPR-Cas9 strategy and PCR. PCR using three sets of outer primers and one set of inner primers verified correct strain composition (Figure S1).

### 3.3. Contribution of C. albicans PAL1 to Growth and Viability

**Figure 2:**
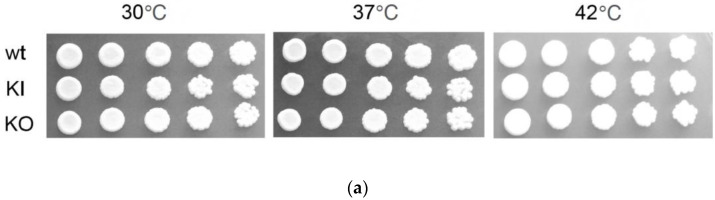

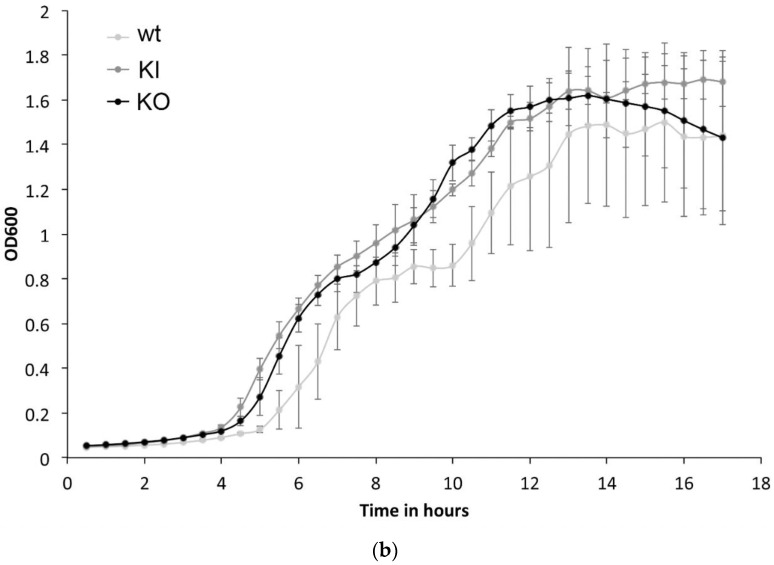
The *C. albicans pal1*∆/∆ null mutant (KO) does not demonstrate impaired growth nor altered cell morphology. (**a**) Growth on yeast extract peptone dextrose (YPD) plates at 30 °C, 37 °C, and 42 °C. The KO does not display any significant reduction in growth compared to the wild-type (WT) and re-integrant (KI) strains. All three temperatures showed similar levels of growth, indicating an absence of temperature sensitivity. (**b**) Growth curve in liquid yeast nitrogen base (YNB) at 30 °C. OD_600_ values were recorded every 30 min for 16 h. Experiments were conducted in triplicate, with three replicates per strain. Error bars indicate the 95% confidence interval of OD_600_ values at each time point for each strain. The doubling times for the WT, KI, and KO strains were calculated as 3.28 ± 0.27 h, 3.16 ± 0.08 h, and 3.37 ± 0.29 h, respectively.

We then examined the growth of the *C. albicans pal1*Δ/Δ mutant in comparison to the control WT and KI strains. Growth on yeast extract peptone dextrose (YPD) agar plates at 30 °C, 37 °C, and 42 °C was similar between the KO and the control strains and demonstrated no temperature sensitivity (Figure 2a). When grown in liquid YPD media at 30 °C, a slight possible growth delay was observed in the KO compared to the KI after 14 h of the growth curve until the end at 17 h, although this is most likely due to settling of cells in the microtiter plate (Figure 2b). In any case, the KO growth in prior hours aligned closely with KI growth and the final levels of KO growth did not dip below final WT growth levels. The WT, KI, and KO strain doubling times were measured as 3.28 ± 0.27 h, 3.16 ± 0.08 h, and 3.37 ± 0.29 h, respectively. The differences between the KO doubling time and those of the WT and KI are not statistically significant (*p* = 0.714, *p* = 0.293). Our results suggest that *C. albicans PAL1* does not play a central role in cell growth.

### 3.4. C. albicans PAL1 Affects Stress Tolerance

**Figure 3:**
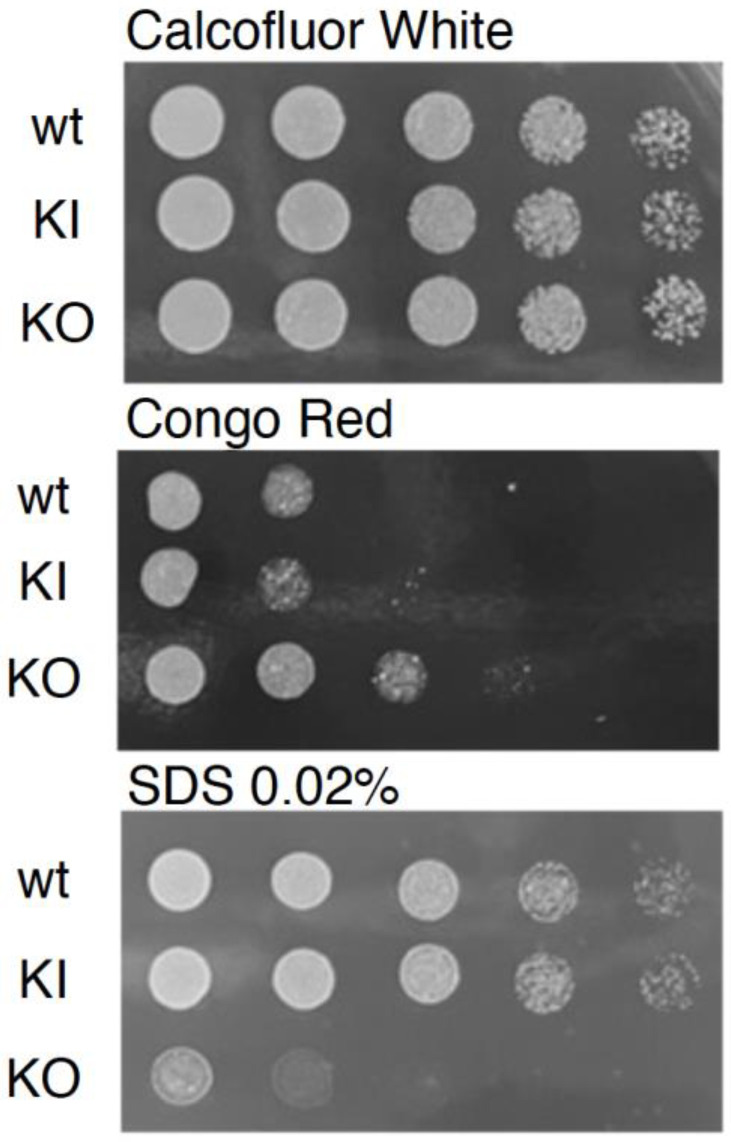
The *C. albicans pal1*Δ/Δ mutant strain (KO) altered stress tolerance. As shown by the plate assays, the null mutant demonstrated increased sensitivity to SDS, which permeabilizes cell membranes. Reduced sensitivity to Congo Red, which disrupts fungal cell walls, was observed in the KO compared to the wild-type (WT) and re-integrant (KI) strains. The KO grew comparably to the WT and KI under Calcofluor White conditions.

We next assessed the contribution of *PAL1* to cell wall integrity by characterizing the growth of the *pal1*Δ/Δ mutant in response to various cell wall stressors and antifungal agents. No growth defect was observed on the medium containing Calcofluor White. However, there was a marked reduction in growth for the null mutant compared to the WT and KI control strains on media containing 0.02% sodium dodecyl sulfate (SDS), which permeabilizes cell membranes (Figure 3). In contrast, there was a reduction in growth inhibition of the KO strain when compared to the WT and KI strains on plates with Congo Red, a compound that disrupts fungal cell walls (Figure 3).

**Figure 4:**
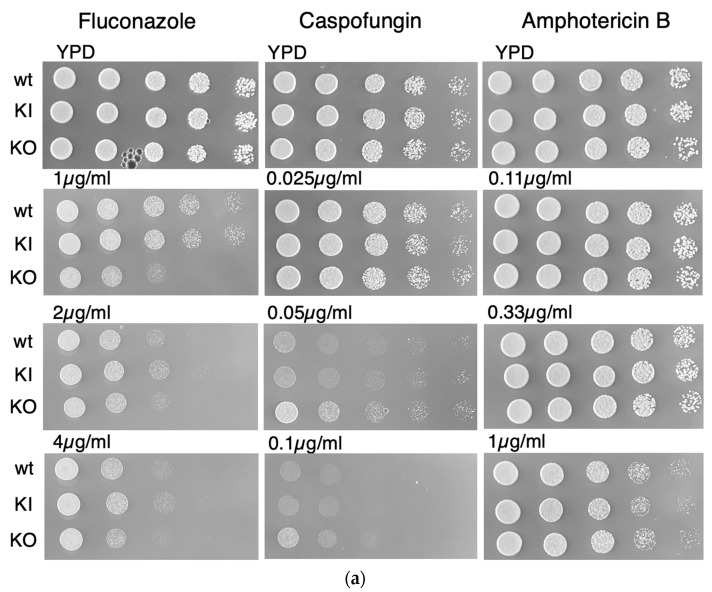

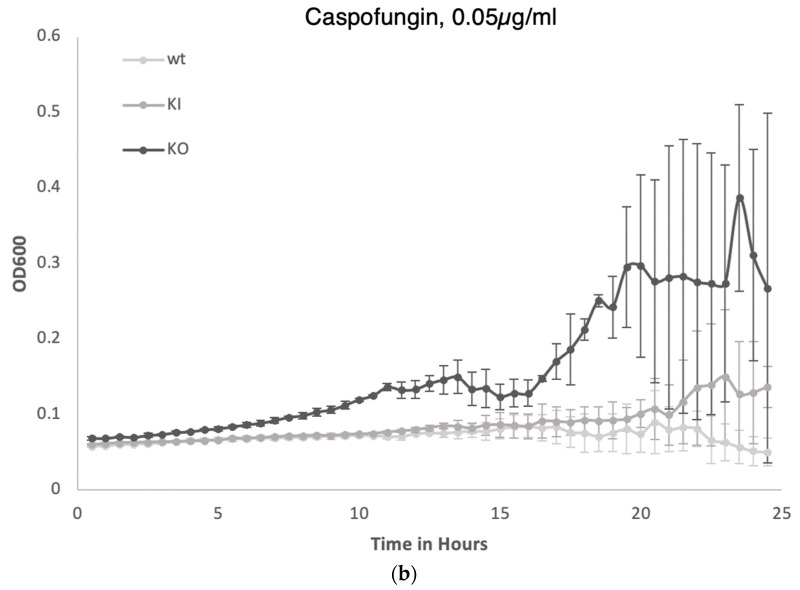
The *C. albicans pal1*Δ/Δ mutant strain (KO) shows reduced sensitivity to the antifungal drug caspofungin compared to the wild-type (WT) and re-integrant (KI) strains. (**a**) As demonstrated in the plate assays, the KO strain exhibits better growth than the WT and KI strains in media containing caspofungin. However, the KO strain grew comparably to the WT and KI in all concentrations of antifungal drug fluconazole and amphotericin B. (**b**) The KO strain also exhibits reduced susceptibility compared with the WT and KI strains in a liquid YPD medium assay with caspofungin at a concentration of 0.05 µg/mL.

When grown on media containing the three common antifungal drugs fluconazole, caspofungin, and amphotericin B, the *pal1*Δ/Δ null mutant exhibited no change in sensitivity to fluconazole (Figure 4). In contrast, a mild reduction in growth impairment in the null mutant was observed on YPD agar plates with 0.1 µg/mL caspofungin compared to controls, indicating reduced susceptibility to this agent. All three (WT, KI, KO) strains grew at all three concentration levels under each drug condition. Reduced susceptibility to caspofungin was also demonstrated in a liquid YPD medium assay with 0.05 µg/mL caspofungin for the null mutant, while liquid media assays with 0.2 µg/mL fluconazole and 0.33 µg/mL amphotericin B demonstrated no changes in sensitivity for the null mutant compared to the WT and KI strains. Caspofungin disrupts cell wall integrity by inhibiting glucan synthase in contrast to fluconazole [35], which interferes with ergosterol synthesis to decrease membrane stability [36]. The decreased susceptibility of the KO strain to the cell wall active agents caspofungin and Congo Red suggests that the *C. albicans PAL1* gene plays a complex role in the maintenance of cell wall integrity. The increased sensitivity of the KO strain to SDS indicates a role of *PAL1* in contributing to maintaining the cell membrane.

### 3.5. Membrane-Related Endocytosis Is Intact in the C. albicans pal1Δ/Δ Mutant

To investigate whether there were any defects in endocytosis and intracellular membrane trafficking in the *pal1*Δ/Δ mutant, we evaluated the intake of the lipophilic fluorescent dye FM4-64. After FM4-64 enters into the outer membrane, it is internalized through clathrin-based endocytosis, which allows visualization of the endocytic transport process towards the vacuole via intermediate compartments. 

**Figure 5:**
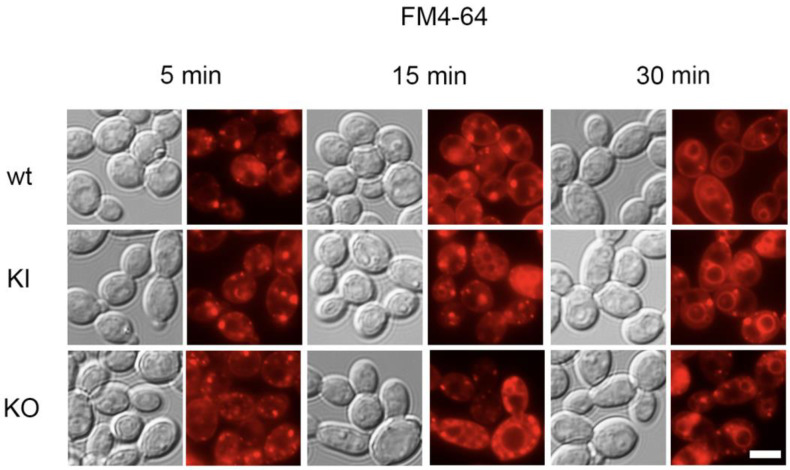
Membrane-related endocytosis is unaffected in the *C. albicans pa11*Δ/Δ mutant (KO). Membrane-related endocytosis was observed over time using lipophilic dye FM4-64 (red), then visualized using DIC and fluorescence microscopy. At 5 and 15 min after incubation at room temperature, the dye was observed to move from the cell periphery towards the vacuole. At 30 min after incubation at room temperature, the dye was accumulated in the vacuolar membrane in all three strains, as evidenced by the presence of a fluorescent vacuolar “ring”, indicating a lack of active endocytosis delay in the KO mutant. The scale bar is 10 μm.

We observed no delay in endocytosis in the *pal1*Δ/Δ mutant. After 30 min, the FM4-64 stain was observed in the vacuolar membrane of all three strains, which demonstrates that FM4-64 was endocytosed to the vacuole by the KO strain at a similar efficiency level as the control strains (Figure 5). These results are consistent with previous findings that the KO strain did not display altered susceptibility when grown in media containing fluconazole, whose effects depends on cellular internalization [33] (Figure 4). The lack of any observed endocytic defects suggests that *C. albicans PAL1* is not required for endocytosis of FM4-64.

### 3.6. C. albicans PAL1 Is Required for Wild-Type Filamentation

**Figure 6:**
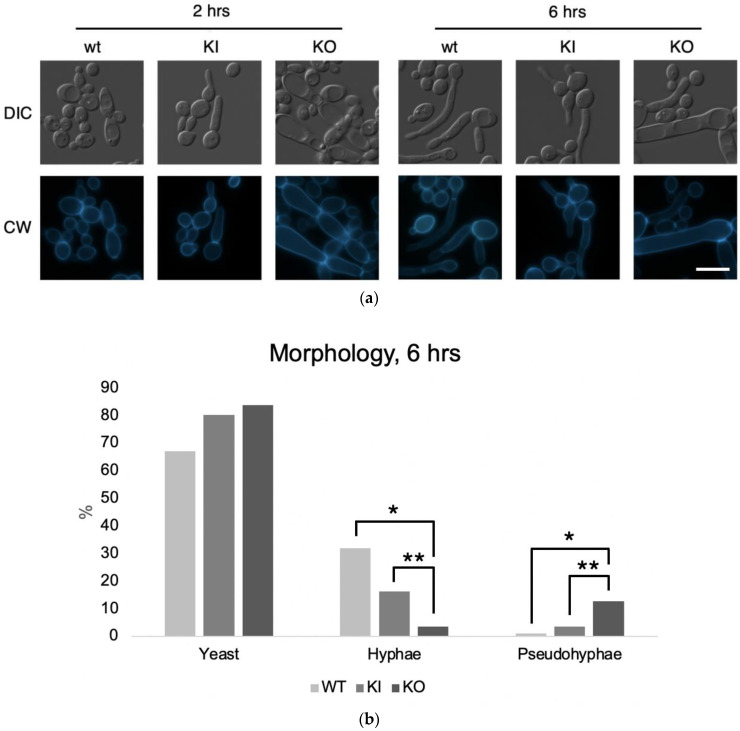

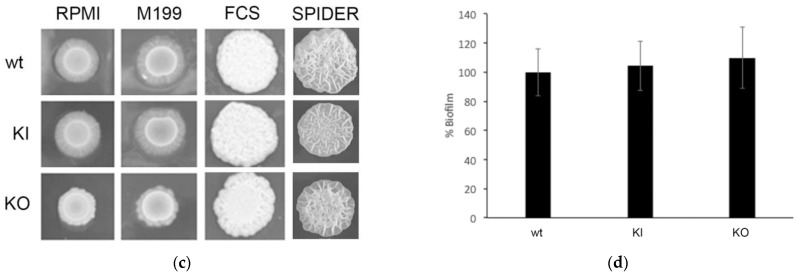
The *C. albicans pal1*Δ/Δ mutant strain (KO) is defective in filamentation but not biofilm formation. (**a**) Filaments visualized under differential interference contrast (DIC) and fluorescence microscopy with Calcofluor White (CW) staining after 2 and 6 h of liquid RPMI-1640 incubation after overnight growth in YPD. While wild-type (WT) and re-integrant (KI) filaments demonstrated hyphal morphology, the null mutant formed pseudohyphae with abnormal septal junctions. The scale bar is 10 μm. (**b**) Proportions of yeast, hyphae, and pseudohyphae present in each of the WT, KI, and KO strains assessed for cells incubated for 6 h in RPMI-1640. Statistical significance was determined using a generalized linear model (GLM) with a least-square means estimation of difference. Compared to the WT and KI strains, the KO strain contained a significantly greater proportion of pseudohyphae and a significantly lower proportion of hyphae (*p* < 0.0001 for all). Significant differences between the WT and KO strains are marked with *. Significant differences between the KI and KO strains are denoted by **. (**c**) Agar plate assays of *C. albicans* hyphal formation. WT, KI, and KO strains were spotted on filamentation-inducing media (RPMI-1640, M199, FCS, and Spider) and incubated at 37 °C for 72 h, then photographed. Under RPMI-1640 and M199 conditions, the KO exhibited a substantial reduction in filamentous growth around the spotted colony, suggesting impaired filamentation. (**d**) Biofilm metabolic activity measured using an XTT reduction assay. Error bars indicate standard deviation. Statistical significance was determined with Student’s *t* test (WT/KO *p* < 0.0001; KI/KO *p* < 0.0001; WT/KI *p* = 0.07). Relative to the WT and KI strains, the biofilm activity in the KO was not significantly lower, indicating a lack of defect in biofilm formation in the *C. albicans pal1*∆/∆ null mutant.

As proper *C. albicans* hyphal and biofilm formation require intact cell wall and remodeling processes, we next investigated whether the defects in the cell wall stress response also impact filamentation. Using solid filamentation media, including RPMI-1640, Medium 199 (M199), fetal calf serum (FCS), and Spider media, we assessed the ability of the *pal1*Δ/Δ mutant to successfully form hyphae (Figure 6c). On RPMI-1640 and Medium 199 agar plates, we saw that filamentous growth was reduced in the KO compared to the WT and KI controls, which had robust filamentous growth on and around the spotted colony. In liquid RPMI-1640 media, the null mutant showed primarily pseudo-hyphal growth with abnormal septin ring formation and septin rings at cell junctions in the KO strain that are more constricted compared to those of the WT and KI strains (Figure 6a), whereas the control stains exhibited true hyphal growth (Figure 6b). At 6 h, the proportions of WT or KI cells that were hyphae were significantly greater than the proportion of KO cells that were hyphae (*p* < 0.0001 for both). The proportions of WT or KI cells that were pseudohyphae were significantly less than the proportion of KO cells that were pseudohyphae (*p* < 0.0001 for both).

To investigate any effects of defective filamentation on the ability to form biofilms, we evaluated biofilm metabolic activity. Relative to the WT and KI controls, biofilm metabolic activity was not significantly different in the KO strain (Figure 6c). These results together demonstrate that *C. albicans PAL1* is required for proper hyphal growth and formation yet has a negligible impact on biofilm metabolic activity.

### 3.7. Dissolution of Cell–Cell Adhesions Is Unaffected in the C. albicans pal1Δ/Δ Mutant

Because extracellular protease secretion is a key virulence-related attribute in *C. albicans* along with hyphal and biofilm formation, we analyzed whether there were defects in protease secretion in the null mutant. Given the filamentation defects in the *pal1*Δ/Δ mutant, we investigated their effects on virulence-associated traits using a human vaginal keratinocyte (VK-2) model of infection. *C. albicans* virulence is partially aided by the secreted aspartic proteases Sap4 to Sap6 that digest E-cadherin, a mammalian adhesion protein present at tight junctions. These proteases support the ability of *C. albicans* to target tight junctions between host cells to disrupt cell–cell adhesions [37,38]. 

**Figure 7:**
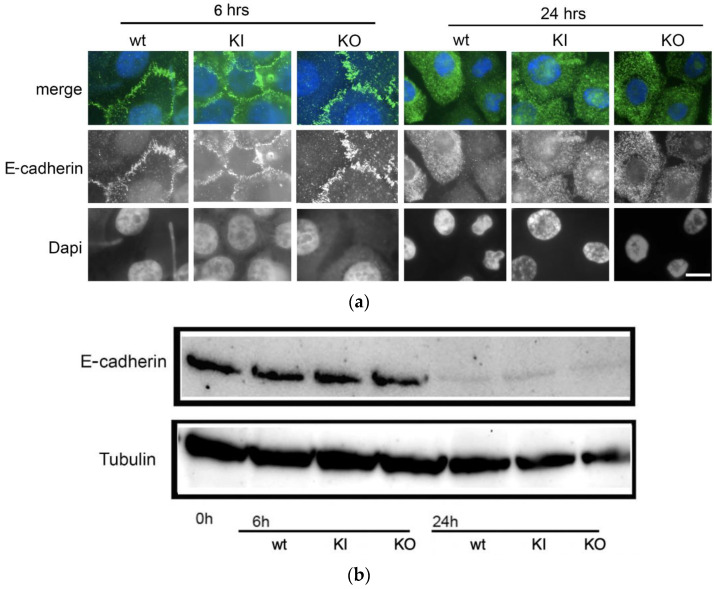
The ability to dissolve cell–cell adhesions in human VK-2 cells remains unaffected in the presence of *C. albicans pal1*Δ/Δ null mutant (KO). (**a**) Human VK-2 cells were infected with wild-type (WT), re-integrant (KI), and KO strains then incubated for 6 and 24 h. E-cadherin was fluorescently labeled using a GFP-tagged antibody and observed as punctate structures present at epithelial cell junctions. DAPI dye was used to label the nucleus, and the merged E-cadherin and DAPI images are displayed in the top row. E-cadherin-labeled junctions were degraded at comparable levels after 24 h by all *Candida* strains. The scale bar is 10 μm. (**b**) Western blot of E-cadherin in *C. albicans*-infected VK-2 cells. E-cadherin is absent in the WT-, KI-, and KO-infected VK-2 cells 24 h post infection, indicating a lack of defect in the ability to disrupt host cell–cell junctions in the *C. albicans pal1*Δ/Δ null mutant. Tubulin was used as a loading control.

For 6 h and 24 h after VK-2 cells were infected with *C. albicans* WT, KI, or KO strains, we visualized the labeled E-cadherin proteins with fluorescence and DAPI microscopy. The labeled tight junctions appeared intact 6 h after infection with all three *C. albicans* strains, but no E-cadherin staining was observed in VK-2 cells infected with any strain after 24 h (Figure 7a). Western blotting to visualize impaired dissolution of cell–cell adhesions for the null mutant was also used to determine whether E-cadherin was present in VK-2 cells at 0-, 6-, and 24 h post infection (Figure 7b). E-cadherin levels were similar in VK-2 cells infected with WT, KI, and KO strains at 6 h. After 24 h, E-cadherin was virtually undetected in cells infected with either of the three strains, which aligns with the microscopy results. These findings indicate that *C. albicans PAL1* deletion is not associated with any changes in the virulence-associated ability to damage cell–cell adhesions in host VK-2 cells.

### 3.8. Ability to Kill Host Cells Does Not Diminish in the C. albicans pal1Δ/Δ Mutant

**Figure 8:**
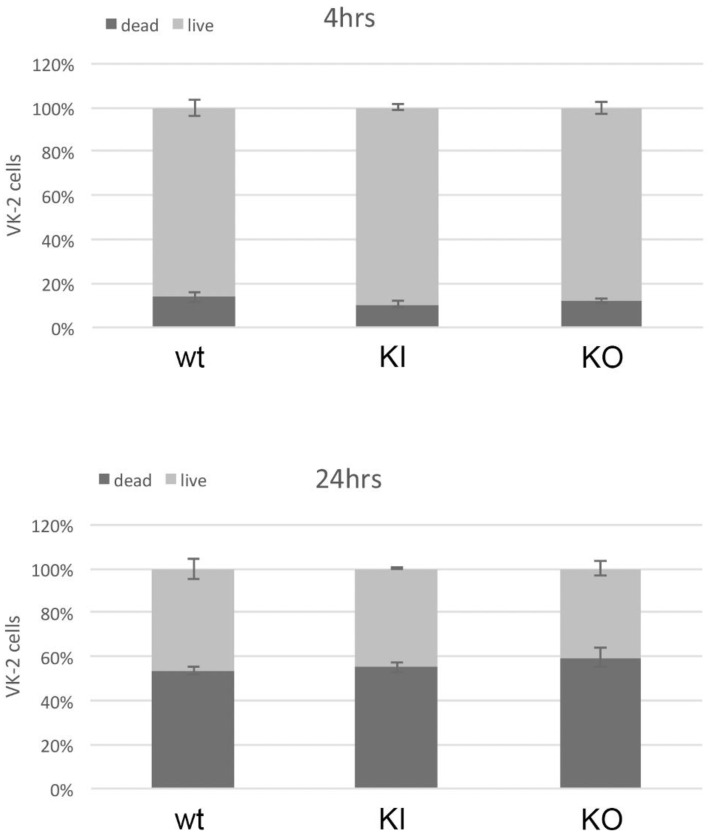
The *C. albicans pal1*Δ/Δ mutant demonstrates a similar level of ability to kill human VK-2 cells in vitro. Proportion of live (white bars) and dead (black bars) VK-2 cells 4 h and 24 h after *C. albicans* infection. Error bars represent the standard deviation. While 50% of cells infected with the wild-type (WT) strain and 55% of cells infected with the re-integrant (KI) strain were dead after 24 h, nearly 60% of the cells infected with the KO strain were dead as well, suggesting that the capacity to kill host cells is unaffected in the KO strain.

Given the ability of *C. albicans* to kill host cells in addition to disrupting tight junctions, we further investigated the role of *PAL1* in *C. albicans* pathogenesis using a microplate Live/Dead assay (Invitrogen, Waltham, MA, USA). We measured VK-2 cell growth 4 and 24 h after infection with WT, KI, or KO strains. At 4 h post infection after exposure to any of the three strains, almost 100% of VK-2 cells were still alive (Figure 8a). At 24 h, the WT and KI control strains, respectively killed about 50% and 55% of VK-2 cells, whereas nearly 60% of the VK-2 cells infected with the KO strain were killed (Figure 8b). The KO strain was just as effective and timely in killing VK-2 cells in vitro as the WT and KI. Thus, *C. albicans PAL1* does not directly contribute to host cell death in this model.

## 4. Discussion

Previous studies on early endocytosis genes in *C. albicans* such as *ENT2* and *END3* have suggested that they play a role in pathogenesis through their involvement in clathrin-mediated endocytosis (CME) in *C. albicans* [2,9,10]. Although several CME coat protein genes have been characterized, others are yet to be studied. In this work, we focused on the *C. albicans* ortholog (*C3_01890C*) of *S. cerevisiae PAL1* (*YDR348C*) and its contribution to growth, stress tolerance, filamentation and biofilm formation, and virulence. The two highly homologous alleles of *PAL1* contain only three differences between the 1332 nucleotides that lead to one silent and two similar amino acid substitutions.

An analysis of structural homology with *C. albicans C3_01890C* and *S. cerevisiae PAL1* was conducted through sequence alignment, which found a moderate level of alignment and several regions of conservation. Further comparisons with ten other fungal species, including eight other *Candida* species (three of which are shown in Figure 1), also found notable regions of cross-species conservation. Because proline side chains produce a kink in the peptide backbone due to their rigidity, the absolute conservation of seven prolines (P) across all species may be notable for protein structure and may support thermal stability. At least one of the two small NPF motifs, known to bind EH domains, are found in all the *PAL1* orthologs examined, indicating the integration of *PAL1* in the endocytic pathways. 

Next, we did not detect a growth defect or temperature sensitivity for the *C. albicans* pal1Δ/Δ mutant (KO). Because *C. albicans* growth depends on adapting to external nutrient conditions [39], the absence of any growth delays indicates that the intracellular nutrient trafficking pathways under standard nutrient conditions remain comparable to the WT and KI controls [40], or alternatively, compensatory mechanisms overcome any trafficking defects. The absence of growth defects in the KO suggests that *C. albicans PAL1* is non-essential in the processes of nutrient transport and cytokinesis. Nonetheless, the delay in filamentous growth observed in the KO compared to the WT and KI was only seen in RPMI-1640 and Medium 199 agar plates, which are both minimal media plates, and not in the FCS and Spider agar plates, suggesting a possible indirect effect on filamentation. Under these minimal media conditions, filamentous growth in the KO could be indirectly impacted by an impairment in nutrient uptake that is linked to nutrient deprivation.

However, the absence of the *C. albicans PAL1* gene broadly affects both cell membrane and cell wall integrity. The KO exhibited increased sensitivity to the cell-membrane-active agent SDS, indicating that *PAL1* contributes to cell membrane integrity. Because the KO also demonstrated decreased susceptibility to the cell wall stressor Congo Red and the cell wall active drug caspofungin, *PAL1* appears to have a complex role in the response of *C. albicans* to cell wall stressors. The fungal cell wall is generally composed of an inner layer of chitin and β-1,3-glucan covered by an outer layer of cell wall proteins. Caspofungin interferes with fungal cell wall integrity by inhibiting glucan synthase [41], which is responsible for generating β-linked glucans. Congo Red impedes fungal cell wall assembly through interacting with β-linked glucans and chitin [42]. Because both of their mechanisms of action rely on β-linked glucans, *PAL1*′s function in cell wall maintenance likely relates to the relationship between β-linked glucans and the cell wall. In contrast, no significant difference in growth was observed for the antifungal drugs fluconazole and amphotericin B which affect the cell membrane, nor for the chitin synthesis inhibitor Calcofluor White [42]. Fluconazole interferes with ergosterol metabolism to impair plasma membrane integrity [36], and amphotericin B binds ergosterol in the fungal cell membrane, leading to pore formation [43]. Therefore, the role of *C. albicans PAL1* in cell membrane integrity does not appear to be related to the ergosterol synthesis or function. Based on the limited number of studies on *PAL1* in both *C. albicans* and *S. cerevisiae*, the exact mechanisms of these phenotypes have not been clarified. *S. cerevisiae* Pal1 has been identified as an early coat protein in clathrin-based endocytosis (CME) that localizes to endocytic sites of the bud neck. *PAL1*′s role in the *C. albicans* CME pathway may be similar, given the high degree of structural and functional conservation of endocytosis in yeasts and other eukaryotic cells. In contrast to the *pal1*Δ/Δ mutant with unaltered sensitivity for fluconazole, the null mutants (*ent2*Δ/Δ and *end3*Δ/Δ) of the *C. albicans* early endocytosis genes *ENT2* and *END3*, also proposed as CME coat proteins, demonstrated reduced susceptibility to fluconazole. *ENT2* and *END3* also expressed increased sensitivity to a broader collection of drugs and stressors, including amphotericin B and Calcofluor White, although all three null mutants are sensitive to SDS [9,10]. Taken together, these differences indicate that early endocytosis genes occupy distinct roles in the highly orchestrated process of early endocytosis, which has an impact on antifungal drug resistance through as of yet undefined mechanisms.

Additionally, our results indicate that *C. albicans PAL1* is non-essential to clathrin-mediated endocytosis. In contrast, the null mutants of *ENT2* and *END3* demonstrate marked endocytic defects. These differences suggest further distinctions between early-coat proteins such as Pal1 compared with middle- and late-coat proteins, such as Ent2 and End3, respectively [9,10]. Interestingly, although the *pal1*Δ/Δ mutant (KO) demonstrated impaired hyphal growth and septin ring formation under some conditions, its ability to form biofilms was comparable to that of the WT and KI controls. Nevertheless, its tendency to form pseudohyphal filaments under some conditions instead of true hyphae indicates that the *C. albicans PAL1* gene has an impact on hyphal formation. In *C. albicans*, septins are cytoskeletal filament-forming proteins that also regulate hyphal morphogenesis [44,45]. Because hyphal morphology is dependent on forming septin rings at cell junctions [46], *PAL1*′s influence on hyphal formation in conjunction with minimal nutrient conditions suggests that the gene contributes to nutrient utilization or uptake processes required for filamentation. 

Given the lack of a growth defect observed in the *C. albicans pal1*Δ/Δ mutant, the similarity in biofilm metabolic activity cannot be explained by any differences in baseline growth rates. Although the KO exhibits a much greater proportion of pseudohyphae instead of hyphae, its ability to adhere to a solid surface to form a layer of anchoring yeast cells during the “seeding” step of biofilm formation is unaffected by the presence or absence of *PAL1* [47]. Hence, even though *C. albicans PAL1* plays an important role in mediating hyphal formation, these effects do not affect overall levels of biofilm formation. Consistent with their endocytic defects, mutants lacking the middle-coat protein Ent2 or the late-coat protein End3 display both impaired filamentation and biofilm formation [9,10]. Moreover, the *C. albicans* homolog of the *S. cerevisiae* late-coat protein-encoding gene *LAS17*, *WAL1*, appears essential to filamentation and hyphal growth as well [22,23].

Active penetration depends in part on the physical force that is exerted by elongating hyphae in order to produce greater epithelial cell damage in vivo [48]. Despite the pseudohyphal morphology of the *C. albicans pal1*Δ/Δ mutant, the lack of defects in its abilities to kill host cells and to dissolve cell–cell adhesion is consistent with the lack of a defect in biofilm formation. In contrast, *ENT2* and *END3* differ from *PAL1* with findings of decreased tissue invasiveness by their null mutants [9,10]. All the dramatic defects in endocytosis, biofilm formation, and virulence in mutants lacking *ENT2* and *END3* are absent in the *pal1*Δ/Δ mutant, indicating a divergence of function within the CME pathway despite their shared trait as coat proteins functioning during the process of early endocytosis. These studies indicate that while *PAL1* plays an important role in cell wall integrity and filamentation, it is dispensable for biofilm formation and tissue invasiveness in our in vitro models. Intriguingly, loss of *PAL1* led to reduced susceptibility to the cell-wall-active agents caspofungin and Congo Red; studies to elucidate the mechanisms of these phenotypes are planned.

## Figures and Tables

**Table 1:** Primers and probes.

Name	Sequence	Reference
PAL1 inner F primer	caa acc ca acca cgc aat aat	This study
PAL1 inner R primer	gct cac ttc act aac tca ctc tc	This study
PAL1 outer 1F primer	gca acg ccc acg att tta tt	This study
PAL1 outer 1R primer	ccg tca tta tca tct tct ccc a	This study
PAL1 outer 2F primer	tag cat gat gca ctt cca caa c	This study
PAL1 outer 2R primer	cca cag ccg tca tta tca tct t	This study
PAL1 outer 3F primer	ctt cca caa cgg gaa aca agt a	This study
PAL1 outer 3R primer	at tcc cat tca gtc gtg gg	This study
PAL1 outer F/donor KI primer	ctt cac ata tcc ccc gct cc	This study
PAL1 outer R/donor KI primer	acg att tgt tgg tgc cct tac	This study
PAL1 KO donor U.95	cac ttc ttc caa tgt acc aac aac cat caa atc att cat att cgt gaa aag gga taa gac aaa acg att taa gac aat tca aac acc aaa taa gg	This study
PALI KO donor L.95	cct tat ttg gtg ttt gaa ttg tct taa atc gtt ttg tct tat ccc ttt tca cga ata tga atg att tga tgg ttg ttg gta cat tgg aag aag tg	This study
PAL1 guide RNA oligo	cgt aaa cta ttt tta att tgc att cat caa aat tat tgc ggt ttt aga gct aga aat agc	This study
AHO1098	caa att aaa aat agt tta cgc aag	[27]
AHO1099	gtt tta gag cta gaa atg caa gtt	[27]

**Table 2:** Plasmids.

Name	Description	Reference
pADH137	C.alb LEUpOUT CAS9 expression plasmid	[27]
pADH118	C.alb LEUpOUT “blank” gRNA plasmid	[27]
pCRPal1	C.alb LEUpOUT “blank” gRNA plasmid with Pal1 guide RNA	This study

**Table 3:** *C. albicans* strains.

Name	Description/Genotype	Reference
AHY940	SC5314 *LEU2* heterozygous knockout a/*α leu2*Δ/*LEU2*	[27]
CRMYPAL1KO	SC5314 *pal1* homozygous knockout a/*α pal1*Δ/*pal1*Δ	This study
CRMYPAL1KI	SC5314 *PAL1* homozygous knock-in a/*α pal1*Δ/*pal1*Δ *PAL1*/*PAL1*	This study

## Data Availability

The data presented in this study are available upon request from the authors.

## Supplementary Materials

The following supporting information can be downloaded at: https://www.mdpi.com/article/10.3390/jof9111097/s1, Figure S1: *C. albicans pal1*∆/∆ null mutant genotype by PCR of genomic DNA. (a) Map and chart of four sets of outer PCR primers used for confirmation: *Pal1* outer F/R, 1F/1R, 2F/2R, 3F/3R. (b) PCR with outer primers on wild-type (WT), re-integrant strains (KI), and null mutants (KO) genomic DNA. (c) PCR with inner primers on WT, KI and KO genomic DNA, Figure S2: The *C. albicans pal1*Δ/Δ mutant strain (KO) is defective in filamentation when visualized under differential interference contrast (DIC) and fluorescence microscopy.
