# Supplementary figures and images for: The Early Endocytosis Gene PAL1 Contributes to Stress Tolerance and Hyphal Formation in Candida albicans
Source: J Fungi (Basel). 2023 Nov 10;9(11):1097. doi: 10.3390/jof9111097 (PMC10672141; doi:10.3390/jof9111097)

Figure S1

A

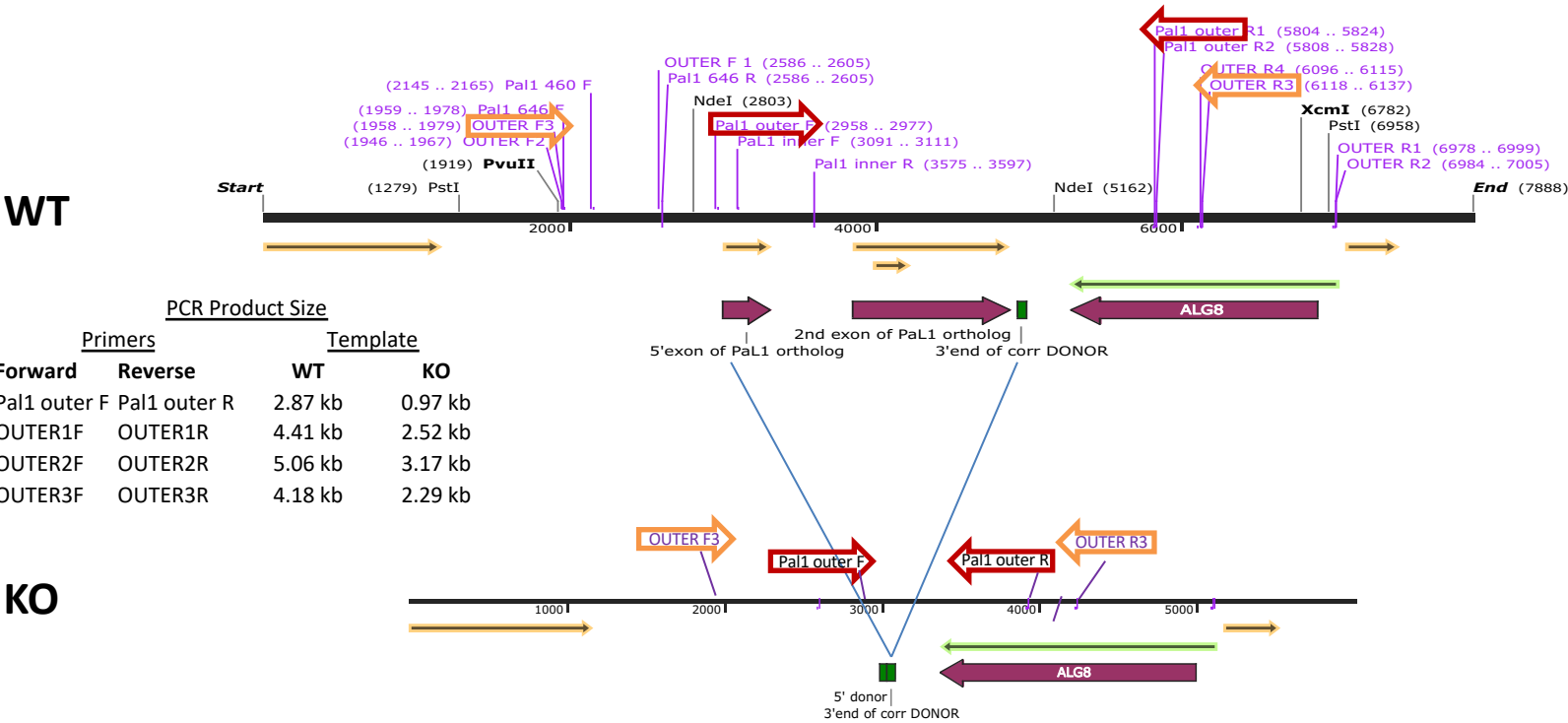

B

## Outer PCR

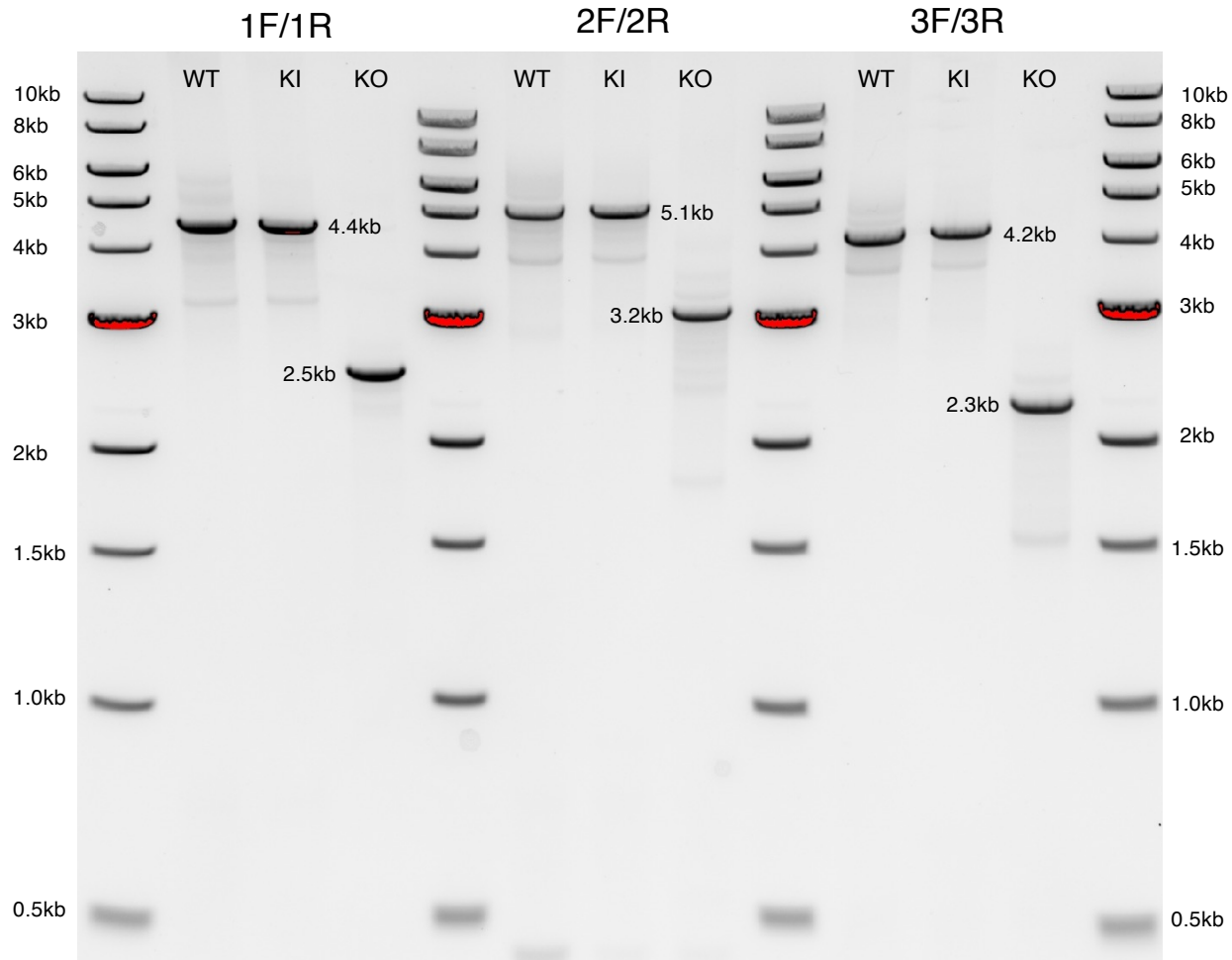

C

Inner PCR

wt    KI    KO

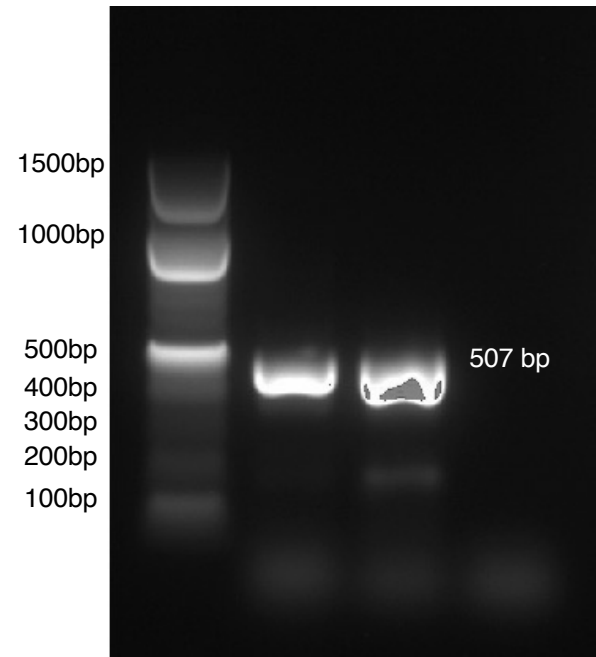

Figure S2

DIC

WT

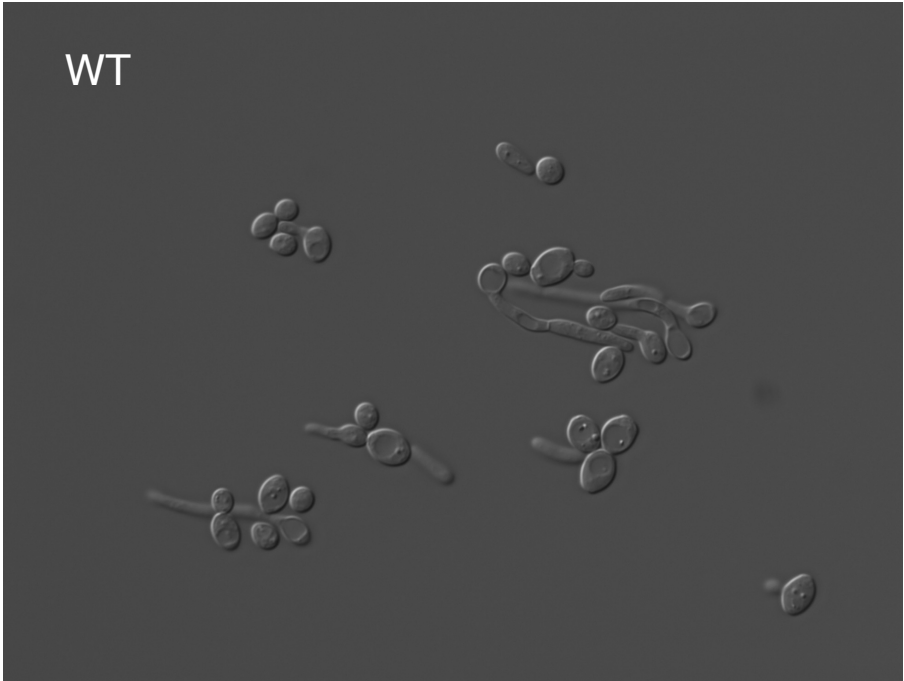

CW

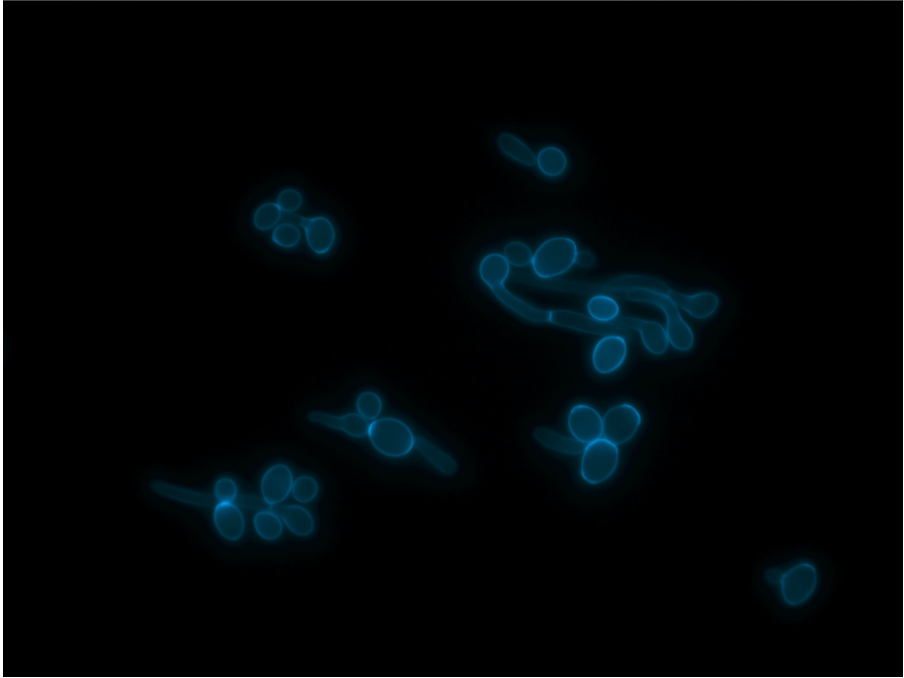

KI

DIC

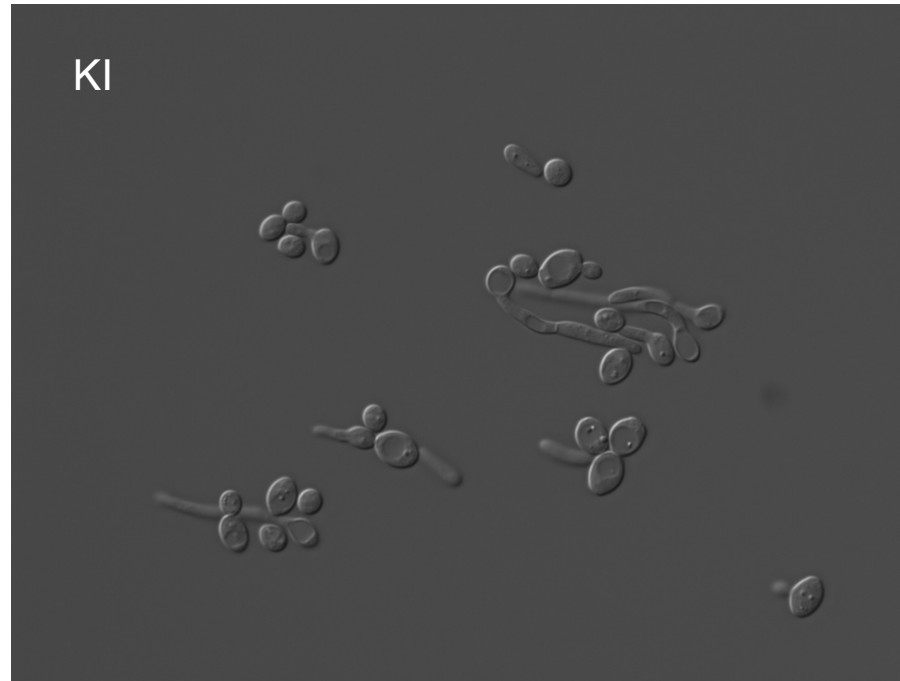

CW

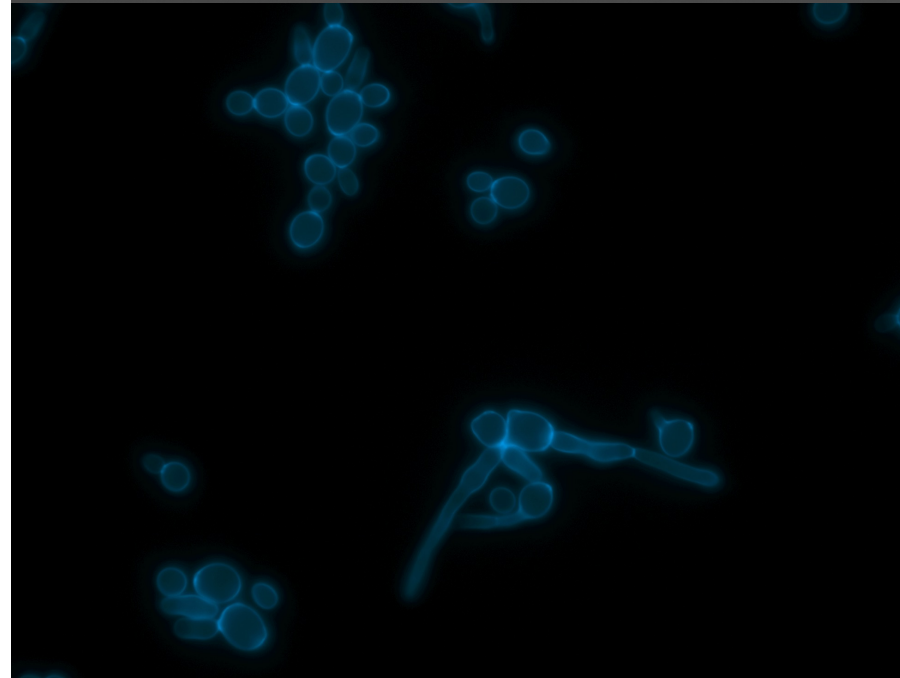

DIC

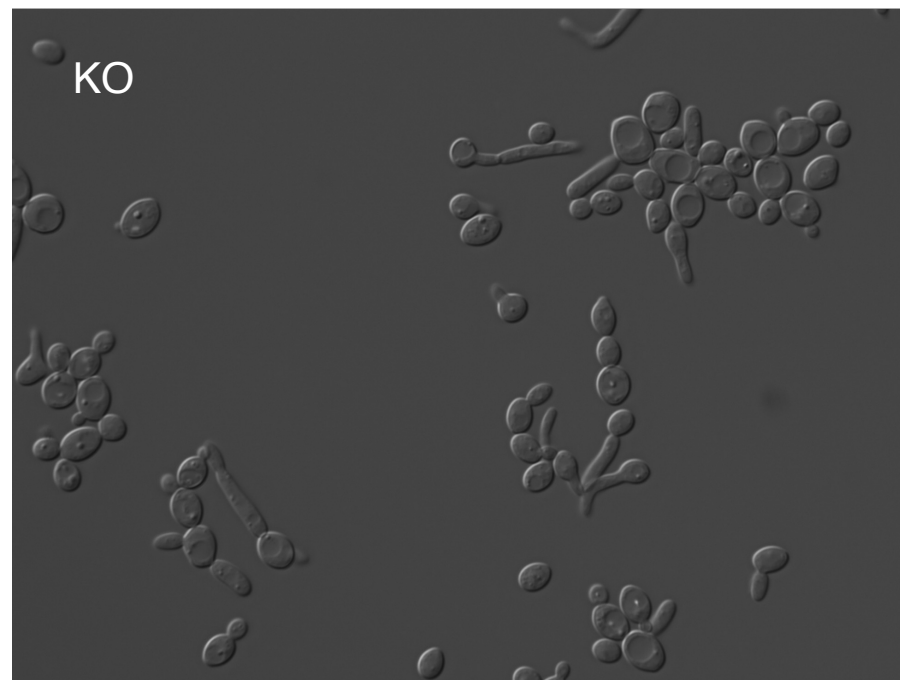

CW

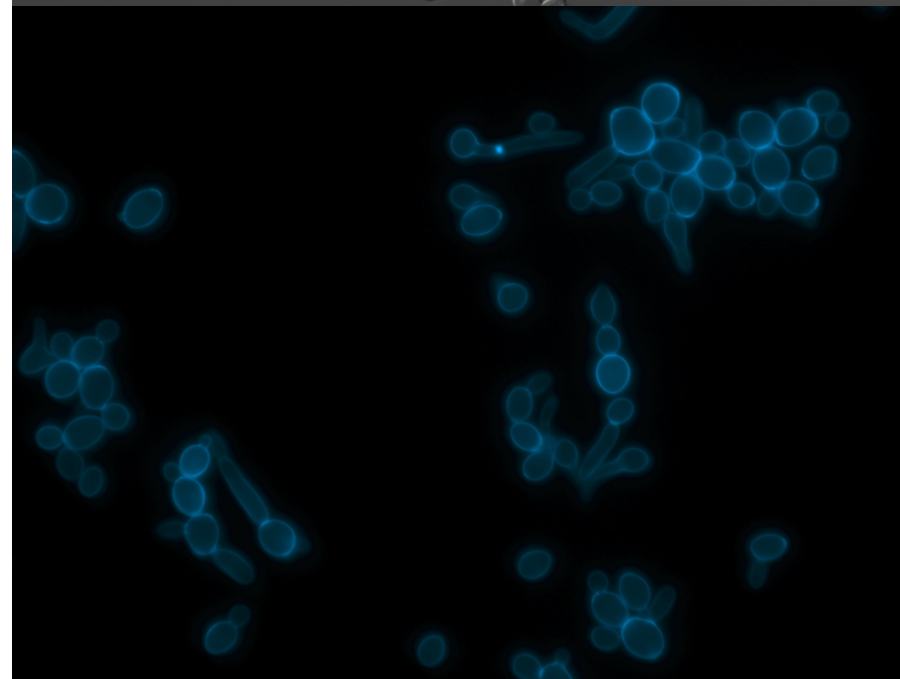

Supplement: Supplementary file 1 [file jof-09-01097-s001.zip › jof-2614768-supplementary.pdf]
